# Prevalence of plasmid-encoded carbapenemases in multi-drug resistant *Escherichia coli* from patients with urinary tract infection in northern Iran

**DOI:** 10.22038/ijbms.2020.34563.8199

**Published:** 2020-05

**Authors:** Mahshid Deldar Abad Paskeh, Mohammad Javad Mehdipour Moghaddam, Zivar Salehi

**Affiliations:** 1 University of Guilan, University Campus 2, Rasht, Iran; 2 Department of Biology, Faculty of Sciences, University of Guilan, Rasht, Iran

**Keywords:** Carbapenemase, Cephalosporines, Multiplex PCR, Plasmid, Resistance

## Abstract

**Objective(s):**

Resistance to carbapenems as the last line for controlling resistant bacteria is increasing due to production of carbapenemase. The aim of this study was to detect the plasmid-encoded carbapenemases using phenotypic methods and multiplex PCR among the multi-drug resistant (MDR) isolates from patients with urinary tract infection (UTI) in northern Iran.

**Materials and Methods:**

Antimicrobial susceptibility testing and extended spectrum β-lactamase (ESBL) production test were performed for 91 MDR *Escherichia coli* strains by disc diffusion and double disk synergy tests (DDST), respectively. Carbapenemases production was confirmed using Hodge test, EDTA double disk synergy test (EDST) and combined disk test (CDT). The isolates were subjected to PCR targeting *bla*_IMP_,* bla*_VIM_, *bla*_KPC_ and *bla*_OXA-48_ β-Lactamase genes.

**Results:**

Resistance of isolates to 1^st^, 2^nd^, 3^rd^, and 4^th^ generations of cephalosporins, carbapenems, and penicillins were 73%, 84.5%, 62%, 37.5%, 17.5%, and 76%, respectively. Based on CDT and Hodge test, 1 (3%) and based on EDST, 2 (6%) of 33 ESBL producers synthesize a type of carbapenemase. The frequency of *bla*_IMP_, *bla*_VIM_, *bla*_KPC_, and *bla*_OXA-48_ genes was 8.7%, 9.8%, 2.1%, and 15.3%, respectively. Existence of* bla*_IMP_ conferred more resistance to cephalotin, fosfomycin, and piperacillin (*P*≤0.01) and carrying *bla*_VIM_ caused more resistance to cephalotin, cefepime, and ceftazidime (*P*≤0.01). The presence of *bla*_KPC_ conferred more resistance to cephalotin and presence of *bla*_OXA-48_ caused more resistance to chloramphenicol and piperacillin (*P*≤0.05).

**Conclusion:**

Identification and controlling of this nearly low frequent ESBL and carbapenemase producing strains are important due to the presence of plasmid genes encoding carbapenemase.

## Introduction

Over the last decade, the emergence of resistance to carbapenems, has become a major public health crisis worldwide especially in developing countries, due to their rapid spread and the lack of development of new antimicrobial agents (1-3). Resistance to carbapenems was reported in 86% of Gram-negative bacterial strains in Iran in 2010 (4).

Throughout 2006–2018, incremental trend of resistance to carbapenems was evident in Iran (5). The least rate of resistance was reported in year 2010 at Milad Hospital (6). During years 2012–2015, a study evaluated the trend of antibiotic resistance in *Acinetobacter baumannii*. Based on their study results, resistance rate in *A. baumannii *increased from 83% in year 2012 to 96% in year 2015. Also 100% of *A. baumannii* isolates during these years were resistant to carbapenem (7). No specific trend was followed by the other microorganisms’ resistance patterns. Most of the carbapenem-resistant strains were isolated from burn patients, and many studies which were conducted in this group were from Motahari Hospital, Tehran, Iran (5). 

Since 1993, wide varieties of carbapenemases have been recognized that belong to three molecular classes: the Ambler class A, B, and D β-lactamases (3). 

In this investigation, four carbapenemases including IMP, VIM, KPC, and OXA-48 were studied. KPC stands for *Klebsiella pneumoniae* carbapenemase and is a class A β-lactamase that has the ability to hydrolyze penicillins, cephalosporins, and carbapenems. KPC was initially reported from a *K. pneumoniae* strain isolated in North Carolina in 1996 (8).

Class B metallo-β-lactamases (MBLs) are mostly of the Verona integron-encoded metallo-β-lactamase (VIM) and IMP types and, more recently, of the New Delhi metallo-β-lactamases-1 (NDM-1) type (3, 9). MBLs can hydrolyze all β-lactams except monobactam (e.g., aztreonam). Their activity is inhibited by EDTA but not by clavulanic acid (9). 

The IMP-type enzymes, initially reported in 1991 in a *Serratia marcescens* clinical isolate from Japan (2), have now been reported all over the world in Enterobacteriaceae, *Pseudomonas aeruginosa*, and *A. baumannii *(2). The most commonly found class B carbapenemases are of the VIM type, which has been identified in all continents (2). The death rates associated with MBL producers are high (18% to 67%)(10, 11). 

OXA stands for oxacillinase and is a diverse group of β-lactamases classified to class D. Some of OXA β-lactamases additionally have the capability to hydrolyze carbapenems. OXA-48 was first found in a *K. pneumoniae* strain isolated in Turkey in 2001 (12). Its production mediates resistance to penicillins and carbapenems (especially imipenem), but not to cephalosporins. In Iran, OXA-48 was first reported in 2017 in the *Escherichia coli* isolates (13).

Among the uropathogenic bacteria, *E. coli* is predominant in both community and nosocomial urinary tract infection (UTI) (14-16). These resistance patterns have shown large inter-regional variability. Understanding the spectrum and resistance patterns may help guide effective empirical antibiotic therapies and decrease treatment failure and costs. 

Contact precautions and outbreak detections require reliable detection of carbapenemases. However, detection of carbapenemase in Enterobacteriaceae is challenging, because carbapenemase-producing *K. pneumoniae* with low carbapenem MICs have been described in the CLSI or EUCAST-susceptible range. Also, a difference between porin loss coupled with an ESBL or AmpC β-lactamase or carbapenemase is not feasible in carbapenem-resistant isolates alone on the basis of the antibiogram (17). Phenotypic tests such as the modified Hodge test are helpful to detect carbapenemases but have low sensitivity for NDM and low specificity (18). Phenotypic tests based on synergy with EDTA are available for detection of MBL but can produce false-positive outcomes with certain strains and cannot distinguish between kinds of MBL (19). Class A carbapenemases such as KPC can be identified through synergy with boronic acid, but if AmpC β-lactamases are coproduced, false-positive synergy test findings occur (20). Thus, confirmation using molecular analysis is essential.

Due to limited information on carbapenemase in Iran (21), identifying the resistant strains is a major challenge for diagnostic laboratories. The carbapenemases that were surveyed in this study were encoded by plasmids and due to their transfer to other isolates, the purpose of this study was to identify types of carbapenemases using phenotypic methods and to determine the frequency of plasmid genes encoding carbapenemases (IMP, VIM, KPC, and OXA-48) among the MDR isolates causing UTI in northern Iran.

## Materials and Methods

***Bacterial isolates***

Urine samples of the patients (138 samples including 31 male and 107 female specimens with mean age of 43 for male and 41 for female) were collected from appropriate patients in early morning mid-stream using sterile, wide mouthed glass bottles with screw cap tops between May and July 2017. Samples were maintained in an icebox until laboratory analysis. Sample collection and its analysis were no more than one hour apart. The usual bacteriological methods were applied for cultivation, isolation and identification of the strains. Urine samples were cultured on Nutrient Agar, Blood Agar, Eosin Methylene Blue Agar (EMB), and MacConkey agar plates and incubated at 37 ^°^C for 18–24 hr. Urine culture was considered positive, if it contained ≥10^5^ cfu/ml. *E. coli* from positive urine cultures identified by their characteristic appearance on the media, Gram staining reaction, by the pattern of biochemical tests such as catalase, oxidase, ONPG, IMViC tests, lactose fermentation, H_2_S and CO_2_ production, urea hydrolysis, and lysine decarboxylase (22). The isolates were stored at -70 ^°^C in a Tryptic Soy Broth containing 15% glycerol until processing.

***Antibacterial susceptibility testing***

To identify the susceptibility of the isolates to antibiotics, the disc diffusion test was used according to Clinical and Laboratory Standards Institute (CLSI)(23) guidelines; the following antibiotics were utilized: ampicillin (AMP)(10 μg), amoxicillin (AMX)(25 μg), oxacillin (OXA)(5 μg), fosfomycin (FOF)(200 μg), piperacillin (PIP)(100 μg), streptomycin (STR)(10 μg), tetracycline (TET)(30 μg), chloramphenicol (CHL)(30 μg), cefepime (FEP)(30 μg), ceftriaxone (CRO)(30 μg), ceftazidime (CAZ)(30 μg), cephalothin (CF)(30 μg), cefazolin (CFZ)(30 μg), cefotaxime (CTX)(30 μg), cefixime (CFM)(5 μg), cefuroxime (CXM)(30 μg), imipenem (IMP)(10 μg), meropenem (MEM)(10 μg), amoxicillin-clavulanic acid (AMC)(20/10 μg), and ciprofloxacin (CIP)(5 μg). *E. coli *ATCC 25922 and ATCC 35218 were used as the standard strains to control the quality of the applied antimicrobial agents. MDR is defined as resistance to three or more antibiotics.

***Detection of ESBL***

In order to identify ESBL, double disk synergy test (DDST), which depends on comparing the inhibition zone given by CAZ (30 µg) and CAZ-plus-clavulanate (30 µg/10 µg) was used. A difference of ≥5 mm between the zone of CAZ-plus-clavulanate and CAZ alone was taken to indicate ESBLs production as advocated by CLSI (23).

***Hodge test***

Briefly, a 0.5 McFarland bacterial suspension of* E. coli *ATCC 25922 as control or susceptible strain was inoculated on the surface of a Mueller-Hinton agar (MHA) as a lawn culture. After brief drying, a 10 µg imipenem disk was placed at the center, and the test isolate was streaked from the center to the periphery of the plate and the plate was incubated overnight. Isolates which produced a cloverleaf-shaped inhibition zone were recognized as producers of carbapenemase (24).

***Imipenem-EDTA combined disk test (CDT)***

As recommended by CLSI, the control strain was cultured as a lawn on the MHA plate along with test isolates (turbidity of 0.5 McFarland). Then, two 10-μg meropenem discs were located on the lawn culture with 20 mm distance from center to center of the discs. In one of the meropenem disks, a 10 μl 0.5 M EDTA was added and incubated overnight. Isolates indicating a rise of ≥7 mm in the meropenem+EDTA disc’s inhibition zone size compared to the meropenem disc alone were known MBL producers (24).

***EDTA-disk synergy test (EDST)***

An overnight broth culture of the test isolate was suspended to the turbidity of a 0.5 McFarland and used to swab inoculate a MHA. A 10-μg meropenem disc and a blank disk (Whatmann filter paper no. 2, 6 mm in diameter) were located 10 mm apart from edge to edge, 10 μl EDTA solution 0.5 M was then used as the blank disc. The plates were incubated at 37 ^°^C overnight and an expanded inhibition zone was interpreted as positive EDS (24).

***Multiplex PCR technique***

DNA extraction was performed with suspending one colony in 100 µl of distilled water (95 ^°^C for 10 min) followed by centrifugation of the cell suspension. The DNA concentration and purity were determined by spectrophotometric measurement of absorbance at 260 and 280 nm by a UV spectrophotometer. All DNA samples were dissolved in water and stored at -20 ^°^C. The PCR reactions were carried out using a 96-well mini PCR System Thermal Cycler (BioRad, USA) in a final volume of 25 µl containing 200 ng of each primer, 50 ng genomic DNA, 1.5 mM MgCl_2_, 200 µM dNTPs, and 1.0 U of Taq DNA Polymerase in the buffer provided by the manufacturer (25). The sequences of specific primers were designed based on relevant DNA sequences available in the NCBI GenBank database (http://www.ncbi.nlm.nih.gov/genbank) using Oligo-primer analysis software (Version 7.54, Molecular Biology Insights, USA). Primers sequences were listed in Table 1.

Amplification was carried out with the following thermal cycling conditions: 10 min at 94 ^°^C and 30 cycles of amplification consisting of 94 ^°^C for 40 sec, 60 ^°^C for 40 sec, and at 72 ^°^C for 1 min, with 7 min at 72 ^°^C for the final extension. For the multiplex PCR analysis, the annealing temperature was at 55 ^°^C for amplification of *bla*_VIM_, *bla*_IMP_, and *bla*_KPC_ genes, and 57 ^°^C for amplification of *bla*_OXA-48 _gene. The PCR products were subjected to electrophoresis on a 2% agarose gel and observed under UV after staining with ethidium bromide (25). 

***Statistical analysis***

The data were statistically analyzed using One-way analysis of variance (ANOVA) and differences among the means were determined at *P≤0.01* using Duncan’s multiple range tests (by SAS, 9.1).

## Results

***Isolates and their resistance patterns***

**Figure 1:**
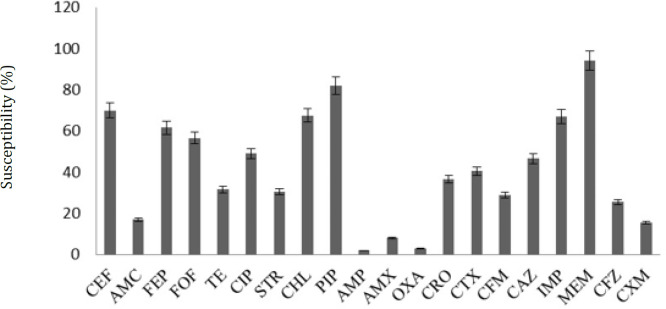
The Susceptibility patterns of various antibiotics against 91 uropathogenic *Escherichia coli* strains isolated from urine samples

From 138 patients that enrolled in this study, 112 urine specimens were infected with bacteria, from which 91 were positive for *E. coli*. The remaining 21 strains included 15 *Enterobacter* spp. isolates, 5 *Proteus mirabilis* isolates and 1 of Group B *Streptococcus* isolates. *E. coli* isolates characterized with distinctive metallic green sheen on EMB and pink colored colonies on MacConkey’s agar, while white or creamy-colored colonies appeared on the nutrient agar. All 91 isolates of *E. coli* had given positive test for catalase, ONPG, lysine decarboxylase, indole, methyl-red test, CO_2_ production, and for lactose fermenting, and negative biochemical test for oxidase, Voges Proskuaer, citrate, urease, and H_2_S production; there we confirmed that these isolates belonged to *E. coli*. It should be noted that the patterns of resistance of these *E. coli* isolates to 20 antibiotics were completely different and therefore all isolates were distinctive. The results of antibiotic susceptibility tests were depicted in Figure 1. The sensitivity of isolates to different antibiotics was different, with meropenem being effective on 91.90%±1.89, piperacillin on 78.94%±3.41, cephalotin on 67.77%±2.20, imipenem and chloramphenicol on 65.34%±1.39 and 64.07%±3.15, respectively, and cefepime on 58.82%±2.37 of the tested isolates. All *E. coli* isolates were identified as MDR bacteria.

***ESBL and carbapenemase detection***

Of total 91 *E. coli* isolates, the synthesis of ESBL was detected in 33 isolates. In this study, phenotypic and genotypic tests were carried out for detection of different types of carbapenemases including IMP, VIM, KPC, and OXA-48. Based on the Hodge test, 3 (3%) of the 33 ESBL-producing *E. coli *isolates produced imipenemase. Using CDT and EDST methods, 1 (3%) and 2 (6%) of the 33 ESBL-producing *E. coli* isolates produced MBL (IMP, VIM, or both and/or other MBLs), respectively. Therefore, these two methods have somewhat different performances in MBL detection. It was interesting that by applying the phenotypic methods in this study, it was proven that none of the non-ESBL-producing *E. coli* isolates were able to produce different types of carbapenemases.

***Multiplex PCR***

**Figure 2:**
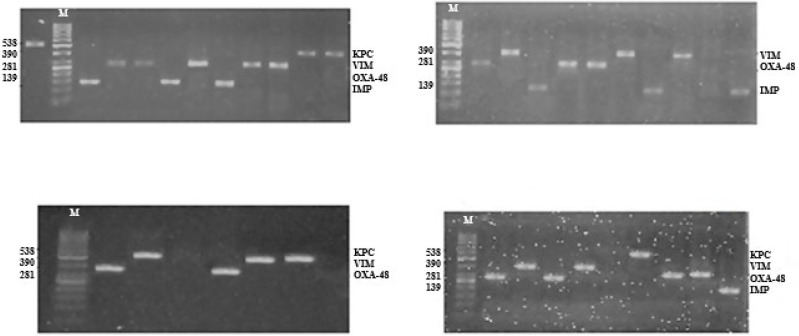
Detection of different carbapenemase genes in some of 33 ESBL-producing *Escherichia coli *isolates by multiplex PCR in 2% agarose gel. In all figures, lane M for molecular marker (50-1000 bp DNA ladder) and other lanes show the amplified fragments of carbapenemase genes including IMP, OXA-48, VIM and KPC

After optimizing the amplification conditions, amplicons with the desired sizes were obtained from the studied isolates and confirmed the specificity of the primers (Figure 2). The results of multiplex PCR analysis showed that frequency of carbapenemaeses genes including IMP, VIM, KPC, and OXA-48 in ESBL-producing *E. coli* isolates were 24% (8/33), 27% (9/33), 6% (2/33), and 42% (14/33) and in all isolates 8.7% (8/91), 9.8% (9/91), 2.1% (2/91), and 15.3% (14/91), respectively. Therefore, *bla*_OXA-48 _and *bla*_KPC_ genes had the highest and the lowest abundance among the *E. coli* isolates, and none of the carbapenemase genes were detected in non-ESBL-producing *E. coli* isolates. Also, the results of phenotypic and multiplex PCR tests were consistent in non-ESBL-producing *E. coli* isolates.

***Carbapenemaeses genes and resistance patterns***

In relation with the simultaneous presence of two or more carbapenemase genes in one isolate, only two strains (1%) included *bla*_VIM_ and *bla*_IMP_ genes and one strain (0.5%) included *bla*_OXA-48 _and *bla*_VIM_ genes. There was no simultaneous presence of *bla*_IMP_ and *bla*_KPC_, *bla*_IMP_ and *bla*_OXA-48_, *bla*_KPC_ and *bla*_OXA-48_, or *bla*_KPC_ and *bla*_VIM_ genes in any of the isolates. Only one isolate (0.5%) carried all four genes studied (Table 2). Existence of IMP conferred more resistance to cefuroxime (100%), cefazolin (87.5%), ampicillin (87.5%), amoxicillin (87.5%), and oxacillin (87.5%)(*P<*0.01), and carrying VIM caused more resistance to ampicillin (100%), amoxicillin (100%), oxacillin (100%), and amoxicillin-clavulanic acid (88.88%)(*P<*0.01). The existence of KPC conferred more resistance to cephalotin (100%) and existence of OXA-48 caused more resistance to ampicillin (100%), amoxicillin (100%), oxacillin (100%), and streptomycin (88.88%)(*P<*0.05). The results of association of two or more carbapenemases genes with the resistance pattern are presented in Table 3.

## Discussion

Resistance to carbapenems is due to carbapenemase and other resistance mechanisms, such as ESBLs, efflux pumps, and/or porin mutations (10). The current emergence of carbapenemase-producing bacteria especially Enterobacteriaceae is of concern because it is often associated with the occurrence of multidrug-resistant isolates, where there are very few drug options available for them, if any (10). Therefore, detection and initial identification of carbapenemase-producing bacteria are important. In some cases, due to the low sensitivity or specificity of phenotypic methods, molecular approaches may also be used (26). Reliable identification of carbapenemases is essential for the implementation of contact precautions and the detection of the outbreak.

In the current study, from the 112 urine specimens from UTI patients infected with bacteria, 91 were positive for *E. coli*. Carbapenems and pipiracillin were the most effective antibiotics and all cephalosporins other than 4^th^ cephalosporin affected more than 50% of isolates. Compared to the ESBL-producing isolates, resistance of non-ESBL-producing isolates was higher than different antibiotics. This is due to another mechanism other than ESBL in resistance to antibiotics. Generally, the frequency of ESBL-producing isolates and the types of carbapenemaese genes among them were low and also in the detection of carbapenemases, there was no correlation between the results of phenotypic and molecular analyses. Due to the similarity of the results of the Hodge and CDT tests, it seems that in the Hodge test, the addition of EDTA did not have any effect on the improvement of the test. Also, the EDST test is comparison with CDT could detect more MBLs and therefore, EDST probably is more reliable. The results of current study in the detection of cabapenemases types in ESBL-producing *E. coli* showed that multiplex PCR is both more sensitive and also more reliable than phenotypic methods due to detection of different carbapenemases and more positive samples.

Given that the resistance of the isolates to imipenem and moropenem was 29% and 6%, respectively, the multiplex analysis identified fewer resistance genes to carbapenems (frequency of *OXA-48*, *KPC*, *IMP*, and *VIM* genes was 7% (10/33), 1% (2/33), 4% (6/33), and 4% (6/33), respectively), which was similar to that of Gheitani and Fazeli (21). The possible reason for this may be the presence of other types of resistance genes to carbapenems that have not been investigated in the current study or other mechanisms of resistance to carbapenem other than carbapenemase production.

Some previous studies in Iran indicated different outbreaks of ESBLs-producing *E. coli*. Contrary to our research, in another study, 115 (89.8%) *E. coli* strains were recognized as ESBL producers (27). Zaniani *et al*. (2012) reported that 43.9% of *E. coli* isolates were ESBL producers (28). Another study identified ESBL-producing *E. coli* in 44.1% and 21.2% of inpatients and outpatients isolates, respectively (29).

Most of the studies have used EDST, CDT, MHT, and E-test for detection of MBL. According to their findings, MBL production varied from 7% to 65%. Some studies recorded the use of EDST as one of the suitable methods to detect Ambler Class B MBL production and the positivity ranged from 14.8% to 72% (16-18). Their results demonstrate that EDST is more reliable and reproducible with elevated positivity rates. In our research like studies of Arakawa, Jesudasan, and John, EDST has shown the highest positivity (30-32).

Similar to previously published data, in the current study low positivity of Hodge test compared with other tests was shown, which varied from 14.8%–56.16% (30-34). Contrary to our study, in most studies, CDT is more robust than EDST and MHT (35). 

Given that phenotypic tests may be false positive and/or low sensitive or specific, confirmation by molecular methods is required. In one study, 183 Enterobacteriaceae were identified from 442 patients with UTI and of them 160 (87.4%) were MDR. The most common isolates were *K. pneumoniae* and *E. coli. *Similar to our study, in their study the prevalence of carbapenemase was a low 2.73% (5/160) and all carbapenemase-producing Enterobacteriaceae (CPE) produced ESBL. In their study, the most effective antibiotic was ciprofloxacin. Significant drug resistances were detected among CPE compared to other MDR Enterobacteriaceae (36). In another study, detection of IMP carbapenemase in 600 Enterobacteriaceae clinical isolates was determined by the PCR method. The most common isolate was *E. coli* 52% (315/600) and the highest rates of resistance were towards ertapenem and imipenem. In combined disk tests by using of the ertapenem or imipenem, 25 isolates were screened positive. Unlike our study, the *bla*_IMP_ gene was not detected in any of the 25 isolates (37).

From a total of 50 carbapenem-resistant *E. coli* isolates in a study, the highest resistance rate was detected to ceftazidime (100%), tetracycline (88%), and amoxicillin (100%). Contrary to our study, the frequency of the *bla*_IMP_ gene was greater (16%), and the *bla*_VIM_ gene was not detected in any of their studied isolates. The prevalence of the *bla*_OXA-48 _gene was almost the same as our study (8%) (38). The disk diffusion test in research on 160 *E. coli* isolates showed that the highest resistance rates were against cefotaxime (20%) and ceftazidime (17%) and the lowest was to tetracycline (1%). Unlike our study, only five isolates (3%) were detected as resistant to imipenem and all five imipenem-resistant strains were confirmed positive for MBL enzymes based on the combination disk diffusion test (CDDT), but PCR did not detect any *bla*_IMP_ or *bla*_VIM_ genes in MBL-producing strains (39).

According to the study by Gheitani and Fazeli (2018) on 183 *K. pneumoniae *isolates, the highest and lowest rates of resistance were detected against cefotaxime (98.2%) and gentamicin (43.6%), respectively. Among the 183 isolates, 134 (73.2 %) were positive based on MHT. Also, in accordance with our study, the prevalence of *bla*_VIM_, *bla*_IMP_, and *bla*_KPC_ genes were low (21).

In a different study, 111 CPE were isolated from different clinical samples. Fifty isolates (55%) were resistant to imipenem and/or meropenem. All the study isolated exhibited a positive MHT. MBL screen test using EDTA and KPC screen test using phenylboronic acid were positive in 54 and 36 isolates, respectively. By using multiplex PCR, carbapenemase-encoding genes were detected in 63 isolates including 58 NDM, 1 VIM, 2 OXA-181, and 6 both NDM and VIM (40). In this study, similar to our study, the prevalence of OXA-48 and VIM carbapenemas was low.

Research showed out of the 100 carbapenem resistant isolates (*E. coli* (25), *K. pneumoniae* (35), *P. aeruginosa* (18), and *A. baumannii* (22)), 70 isolates were MHT positive, while 65 isolates were CDT positive. In five isolates which were MHT positive but CDT negative, none of the 4 genes including *bla*_NDM-1_, *bla*_VIM_, *bla*_IMP,_ and *bla*_KPC_ were detected. The results of the multiplex PCR for four target genes showed only 5 strains with the *bla*_VIM_ gene, 1 strain with the *bla*_KPC_ gene, and none of the strains produced *bla*_IMP_^.^ Out of 100 carbapenem resistant isolates, 65 isolates were harboring one or more than one genes, while in 35 isolates none of the genes was detected. The most common resistance gene was *bla*_NDM-1 _(59/100) followed by *bla*_KPC_ (15/100) while the *bla*_VIM_ gene was least frequent (6/100). Contrary to our research, the *bla*_IMP_ gene did not detect in any of the isolates. Correlation of multiplex PCR with MHT and CDT among carbapenemase-producing isolates is observed (41). 

In the study by Pavelkovich *et al*. in the Baltic States and St. Petersburg, Russia on CPE, of all 9757 strains including 1983 *K. pneumoniae* and 7774 *E. coli* isolated from intensive care patients and different clinical samples, 77 isolates (73 *K. pneumoniae* and 4 *E. coli*)(0.7%) were resistant to carbapenem. In this study unlike our study, of 77 strains, in 15 strains the *bla*_NDM_ gene was detected and in the other 62 strains *bla*_IMP_, *bla*_VIM_, *bla*_GIM_, *bla*_OXA-48_, *bla*_NDM_, or *bla*_KPC_ genes were not identified (42). 

In a recent study, 210 MDR Gram-negative bacilli were obtained from different specimens such as urine (n=108) screened for carbapenemase resistance. They used uniplex PCR for detection of *bla*_NDM-1 _and *bla*_KPC_ genes in *E. coli* and *Klebsiella *and applied multiplex PCR for detection of *bla*_IMP_ and *bla*_VIM_ genes in *P. aeruginosa* and *A. baumannii* isolates. Twenty three (11%) isolates (*E. coli* (6), *K.* *pneumoniae* (3), *P.* *aeruginosa* (5), and *A. baumannii* (9)) were found resistant to meropenem and imipenem by disc diffusion. The results of MHT showed that out of the 23 carbapenem-resistant isolates, 17 (74%) produced MBL. These were further confirmed by the E-test. MHT was negative for all isolates. All 17 isolates were subjected to PCR and found to contain at least one carbapenemase gene. Unlike our study, none of *bla*_KPC_, *bla*_IMP_, or *bla*_VIM _genes were detected in Enterobacteriaceae isolates (43).

In a study on frequency of carbapenemase genes including VIM, IMP, NDM, KPC, and OXA- 48 in 227 MDR Gram-negative bacteria, similar to our study, the most effective antibiotic was meropenem, and 80 strains (35%) were positive for one or more carbapenemase genes. Contrary to our study, IMP-types were the most predominant gene followed by VIM, in 49 (21.59%) and 28 (12%) isolates, respectively. Carbapenemase genes were most detected in *K. pneumoniae* (24, 11%), followed by *P. aeruginosa* (23, 10%), and *E. coli* with 19 isolates (8%)(44).

In one study, 60 bacteria were isolated from urine specimen including 26 strains of *E. coli* and 34 strains of *K. pneumoniae*. It was determined that meropenem and amikacin were the most effective antibiotics on *E. coli,* and imipenem the most effective antibiotic on *K. pneumoniae*. Four *E. coli* and 23 *K. pneumoniae* isolates were positive for carbapenemase production by using the MHT test. Although some results of phenotypic assays matched with the definite PCR identification, some results were misleading. Out of the 29 positive PCR samples, three samples of *K. pneumoniae* were negative for MHT and one *E. coli* sample was MHT positive but negative for PCR. Nine samples were positive for PCR but were determined as carbapenem sensitive by MicroScan (45).

In a study to detect MBL among 100 *A. baumannii* strains, 30 (30%) of the strains were positive for the *bla*_VIM_ gene, but the *bla*_IMP_ gene was not detected in any of the strains (46).

**Table 1:** Group-specific primers used for multiplex PCR (23)

**Gene**	**Primer name**	**Sequence (5ʹ –3 ʹ)**	**Length (bases)**	**Annealing position**	**Product size (bp)**		**Primer concentration**		
*bla*_OXA-48 _like	MultiOXA-48_for MultiOXA-48_rev	GCTTGATCGCCCTCGATT GATTTGCTCCGTGGCCGAAA	18 20	230-247 490-510		281		0.4 0.4
*bla*_IMP_ variants except *bla*_IMP-9_, *bla*_IMP-16_, *bla*_IMP-18_, *bla*_IMP-22 _and *bla*_IMP-25_	MultiIMP_for MultiIMP-48_rev	TTGACACTCCATTTACDG^b^ GATYGAGAATTAAGCCACYCT^b^	18 21	194-211 332-313		139		0.5 0.5
*bla*_VIM_ variants including *bla*_VIM-1 _and *bla*_VIM-2_	MultiVIM_for MultiVIM_rev	GATGGTGTTTGGTCGCATA CGAATGCGCAGCACCAG	19 17	151-169 540-524		390		0.5 0.5
*bla*_KPC-1 _to *bla*_KPC-5_	MultiKPC_for MultiKPC_rev	CATTCAAGGGCTTTCTTGCTGC ACGACGGCATAGTCATTTGC	22 20	209-230 746-727		538		0.2 0.2

bY=T or C; R= A or G; S= G or C; D= A or G or T

**Table 2:** Comparison of the resistance rates between ESBL, non-ESBL-producing, and total *Escherichia coli *isolates against 20 antibiotics and association between carbapenemase genes with resistance pattern among ESBL-producing isolates ^a,b^

**Antibiotic**	**ESBL (n=33)**	**non-ESBL (n=58)**	**Total (n=91)**	**IMP (n=8)**	**OXA-48 (n=14)**	**KPC (n=2)**	**VIM (n=9)**
**CF**	20 (60.60)	13 (22.41)	27 (30)	6 (75)	1 (7.14)	2 (100)	7 (77.77)
**AMC**	21 (63.63)	50 (86.20)	75 (83)	6 (75)	1 (7.14)	0 (0.00)	8 (88.88)
**FEP**	8 (24.24)	24 (41.37)	35 (38.5)	4 (50)	4 (28.5)	0 (0.00)	5 (55.55)
**FOF**	12 (36.36)	26 (44.82)	40 (43.5)	6 (75)	3 (21.42)	1 (50)	4 (44.44)
**TET**	19 (57.57)	41 (70.68)	62 (68.5)	6 (75)	9 (64.28)	1 (50)	2 (22.22)
**CIP**	10 (30.30)	33 (56.89)	47 (51.64)	5 (62.5)	6 (42.85)	1 (50)	5 (55.55)
**STR**	22 (66.66)	41 (69.87)	63 (69.23)	3 (37.5)	12 (85.71)	0 (0.00)	7 (77.77)
**CHL**	3 (9.09)	22 (37.93)	30 (32.96)	2 (25)	7 (50)	0 (0.00)	3 (33.33)
**PIP**	1 (3.03)	13 (22.41)	17 (18.68)	3 (37.5)	4 (28.5)	0 (0.00)	3 (33.33)
**AMP**	30 (90.90)	53 (91.37)	89 (97.80)	7 (87.5)	14 (100)	1 (50)	9 (100)
**AMX**	30 (90.90)	53 (91.37)	84 (92.30)	7 (87.5)	14 (100)	1 (50)	9 (100)
**OXA**	31 (93.93)	56 (96.55)	88 (96.70)	7 (87.5)	14 (100)	1 (50)	9 (100)
**CRO**	18 (54.54)	38 (65.51)	58 (63.73)	5 (62.5)	11 (78.57)	1 (50)	5 (55.55)
**CTX**	12 (36.36)	37 (63.79)	54 (59.34)	6 (75)	12 (85.71)	1 (50)	6 (66.66)
**CFM**	19 (57.57)	43 (74.13)	65 (71.42)	5 (62.5)	11 (78.57)	1 (50)	4 (44.44)
**CAZ**	16 (48.48)	32 (55.17)	49 (53.84)	4 (50)	7 (50)	1 (50)	7 (77.77)
**IMP**	7 (21.21)	20 (34.48)	30 (32.96)	3 (37.5)	2 (14.28)	0 (0.00)	3 (33.33)
**MEN**	5 (15.15)	2 (34.48)	6 (65.93)	0 (0.00)	1 (7.14)	0 (0.00)	0 (0.00)
**CFZ**	18 (54.54)	45 (77.58)	68 (74.72)	7 (87.5)	41.42	1 (50)	6 (66.66)
**CXM**	29 (87.87)	51 (87.93)	77 (84.61)	8 (100)	78.57	1 (50)	7 (77.77)

^a ^Data are presented No. (%), n= number of isolates. ^b ^ESBL= extended spectrum β-lactamase

IMP: imipenem; OXA: oxacillin; KPC: Klebsiella pneumoniae carbapenemase; VIM: Verona integron-encoded metallo-*β*-lactamase

**Table 3:** Association between two carbapenemase genes with resistance pattern in 33 ESBL-producing *Escherichia coli* isolates^a,b^

**Antibiotic**	**IMP and KPC (n=0)**	**IMP and OXA-48 (n=0)**	**IMP and VIM (n=2)**	**KPC and OXA-48 (n=0)**	**KPC and VIM (n=0)**	**OXA-48 and VIM (n=1)**
**CF**	0 (0.00)	0 (0.00)	2 (100)	0 (0.00)	0 (0.00)	1 (100)
**AMC**	0 (0.00)	0 (0.00)	2 (100)	0 (0.00)	0 (0.00)	1 (100)
**FEP**	0 (0.00)	0 (0.00)	1 (50)	0 (0.00)	0 (0.00)	0 (0.00)
**FOF**	0 (0.00)	0 (0.00)	1 (50)	0 (0.00)	0 (0.00)	0 (0.00)
**TET**	0 (0.00)	0 (0.00)	0 (0.00)	0 (0.00)	0 (0.00)	0 (0.00)
**CIP**	0 (0.00)	0 (0.00)	1 (50)	0 (0.00)	0 (0.00)	1 (100)
**STR**	0 (0.00)	0 (0.00)	1 (50)	0 (0.00)	0 (0.00)	1 (100)
**CHL**	0 (0.00)	0 (0.00)	0 (0.00)	0 (0.00)	0 (0.00)	0 (0.00)
**PIP**	0 (0.00)	0 (0.00)	1 (50)	0 (0.00)	0 (0.00)	1 (100)
**AMP**	0 (0.00)	0 (0.00)	2 (100)	0 (0.00)	0 (0.00)	1 (100)
**AMX**	0 (0.00)	0 (0.00)	2 (100)	0 (0.00)	0 (0.00)	1 (100)
**OXA**	0 (0.00)	0 (0.00)	2 (100)	0 (0.00)	0 (0.00)	1 (100)
**CRO**	0 (0.00)	0 (0.00)	1 (50)	0 (0.00)	0 (0.00)	1 (100)
**CTX**	0 (0.00)	0 (0.00)	2 (100)	0 (0.00)	0 (0.00)	1 (100)
**CFM**	0 (0.00)	0 (0.00)	2 (100)	0 (0.00)	0 (0.00)	1 (100)
**CAZ**	0 (0.00)	0 (0.00)	1 (50)	0 (0.00)	0 (0.00)	0 (0.00)
**IMP**	0 (0.00)	0 (0.00)	0 (0.00)	0 (0.00)	0 (0.00)	0 (0.00)
**MEN**	0 (0.00)	0 (0.00)	0 (0.00)	0 (0.00)	0 (0.00)	0 (0.00)
**CFZ**	0 (0.00)	0 (0.00)	2 (100)	0 (0.00)	0 (0.00)	1 (100)
**CXM**	0 (0.00)	0 (0.00)	2 (100)	0 (0.00)	0 (0.00)	1 (100)

^a ^Data are presented No.(%), n= number of isolates

^b ^ESBL= extended spectrum β-lactamases

IMP: imipenem; OXA: oxacillin; KPC: Klebsiella pneumoniae carbapenemase; VIM: Verona integron-encoded metallo-*β*-lactamase

## Conclusion

Although the prevalence of the ESBL-producing strains and the simultaneous presence of several carbapenemase genes in the studied population were nearly low, these low prevalent strains and genes are responsible for resistance to some antibiotics. Thus, identification and controlling of these strains is important due to the presence of plasmid genes encoding carbapenemases and their easy transferability to other clinical isolates.
